# Impact of porin deficiency on the synergistic potential of colistin in combination with β-lactam/β-lactamase inhibitors against ESBL- and carbapenemase-producing *Klebsiella pneumoniae*

**DOI:** 10.1128/aac.00762-24

**Published:** 2024-10-04

**Authors:** Lisa Allander, Karin Vickberg, Elin Fermér, Thomas Söderhäll, Linus Sandegren, Pernilla Lagerbäck, Thomas Tängdén

**Affiliations:** 1 Department of Medical Sciences, Uppsala University, Uppsala, Sweden; 2 Department of Medical Biochemistry and Microbiology, Uppsala University, Uppsala, Sweden; 3 Uppsala Antibiotic Center, Uppsala University, Uppsala, Sweden; University of Fribourg, Fribourg, Switzerland

**Keywords:** *Enterobacterales*, multidrug resistance, combination therapy, synergy, polymyxins, avibactam, ceftazidime, meropenem

## Abstract

Combinations of colistin and β-lactam/β-lactamase inhibitors (BLBLIs) have shown *in vitro* synergy against β-lactamase-producing strains. However, data are limited and conflicting, potentially attributed to variations among the examined strains. This study investigated whether loss of porins OmpK35 and OmpK36 impacts the synergistic potential of colistin in combination with ceftazidime–avibactam or meropenem–avibactam against β-lactamase-producing *Klebsiella pneumoniae*. Genetically modified strains were constructed by introducing *bla*_CTX-M-15_, *bla*_KPC-2_, and *bla*_OXA-48_ chromosomally into *K. pneumoniae* ATCC 35657, in which the major porin-encoding genes (*ompK35*, *ompK36*) were either intact or knocked out. The *in vitro* activity of colistin in combination with ceftazidime–avibactam or meropenem–avibactam was evaluated by time-lapse microscopy screening and in static time-kill experiments. The deletion of porins in the β-lactamase-producing strains resulted in 2- to 128-fold increases in MICs for the β-lactams and BLBLIs. The activity of avibactam was concentration-dependent, and 4- to 16-fold higher concentrations were required to achieve similar inhibition of the β-lactamases in strains with porin loss. In the screening, synergy was observed for colistin and ceftazidime–avibactam against the CTX-M-15-producing strains and colistin and meropenem–avibactam against the KPC-2- and OXA-48-producing strains. The combination effects were less pronounced in the time-kill experiments, where synergy was rarely detected. No apparent associations were found between the loss of OmpK35 and OmpK36 and combination effects with colistin and BLBLIs, indicating that additional factors determine the synergistic potential of such combinations.

## INTRODUCTION

β-lactams are cornerstone antibiotics in the management of severe infections. However, their activity against major Gram-negative pathogens, including *Klebsiella pneumoniae*, is threatened by the emerging extended-spectrum β-lactamases (ESBLs) and carbapenemases. Strains producing β-lactamases often carry a range of other resistance determinants, resulting in multidrug-resistant (MDR) phenotypes with few remaining treatment options (1, 2).

The β-lactamase inhibitors restore the activity of β-lactam antibiotics against MDR *K. pneumoniae* to various extents, depending on which enzymes are produced. Avibactam is active against serine β-lactamases, including ESBLs (e.g., CTX-M-15) and carbapenemases (e.g., KPC and OXA-48) (3, 4). The β-lactam/β-lactamase inhibitor (BLBLI) combination ceftazidime–avibactam has demonstrated clinical efficacy for severe Gram-negative infections (5–8). Although β-lactam resistance in Gram-negative bacteria is largely enzymatic, membrane permeability also plays an important role. Loss of the major porins OmpK35 and OmpK36, which facilitate entry of β-lactams into the cell, has been associated with decreased susceptibility towards β-lactams in *K. pneumoniae* (9–12). Reduced membrane permeability has also been reported to decrease susceptibility to ceftazidime–avibactam (11, 13, 14).

Combination therapy with colistin is a last resort against infections with MDR Gram-negative bacteria (15, 16). Colistin (polymyxin E) targets the lipopolysaccharides and acts by destabilizing the outer membrane, which may allow increased entry of other drugs into the periplasm (15, 17). Based on its mechanism of action, colistin could theoretically act synergistically in combination with BLBLIs, particularly against strains with reduced permeability of β-lactams. However, there is insufficient data to support the clinical benefit of combination therapies with colistin and the new BLBLIs (18, 19). Moreover, *in vitro* studies have generated conflicting results (20–23), possibly attributed to differences in resistance mechanisms (e.g., β-lactamases, reduced permeability, and efflux) in the tested strains.

This study aimed to examine the synergistic potential of colistin in combination with BLBLIs against β-lactamase-producing *K. pneumoniae* and specifically to investigate whether the activity was better against isolates with impaired β-lactam permeability. To test this hypothesis, we created genetically modified *K. pneumoniae* strains encoding commonly encountered β-lactamases (CTX-M-15, KPC-2, or OXA-48) in an otherwise isogenic background with the major porin-encoding genes *ompK35* and *ompK36* either intact or knocked out. The activity of colistin in combination with ceftazidime–avibactam or meropenem–avibactam was evaluated by time-lapse microscopy screening complemented by cell viability testing (spot assay) and in 24-h static time-kill experiments.

## RESULTS

### Whole genome sequencing and antibiotic susceptibility

Whole genome sequencing (WGS) confirmed successful chromosomal integration of β-lactamase genes and deletion of *ompK35/ompK36* where relevant. No mutations of known relevance (SNPs, insertions, or deletions) were detected outside the target regions. The parental *K. pneumoniae* ATCC 35657 strain and all constructs were susceptible to colistin (MICs 0.25 mg/L). The parental strain was also susceptible to ceftazidime (MIC 0.125 mg/L) and meropenem (MIC 0.031 mg/L), and porin loss yielded 2- to 4-fold higher MICs for the β-lactams and BLBLIs (Table 1). The introduction of *bla*_CTX-M-15_ into the parental strain rendered high-level resistance to ceftazidime (256-fold increase in MIC). The introduction of *bla*_KPC-2_ resulted in 8- and 32-fold higher ceftazidime and meropenem MICs, respectively, whereas *bla*_OXA-48_ showed no significant impact on the susceptibility. The deletion of porins in the β-lactamase-producing strains caused additional elevations in MICs: up to 4-fold MIC increases for ceftazidime and ceftazidime–avibactam and 4- to 128-fold higher MICs for meropenem and meropenem–avibactam.

**TABLE 1:** Susceptibilities of *Klebsiella pneumoniae* ATCC 35657 and constructed derivatives (ΔOmpK35/36 and CTX-M-15-, KPC-2-, or OXA-48-producing strains with or without OmpK35/36 porins)^*a*^^,^^*b*^

Strain	MIC (mg/L)						
	CAZ	CAZ-AVI	CAZ-AVI_ratio_	MEM	MEM-AVI	MEM-AVI_ratio_	COL
Parental	0.125 (S)	0.125 (S)	0.25	0.031 (S)	0.016	0.031	0.25 (S)
ΔOmpK35/36	0.5 (S)	0.25 (S)	0.5	0.062 (S)	0.062	0.062	0.25 (S)
CTX-M-15	32 (R)	0.25 (S)	0.5	0.031 (S)	0.016	0.031	0.25 (S)
CTX-M-15 ΔOmpK35/36	128 (R)	0.5 (S)	2	2 (S)	0.062	1	0.25 (S)
KPC-2	1 (S)	0.125 (S)	0.5	1 (S)	0.016	0.062	0.25 (S)
KPC-2 ΔOmpK35/36	2 (I)	0.5 (S)	0.5	16 (R)	0.125	1	0.25 (S)
OXA-48	0.125 (S)	0.125 (S)	0.125	0.062 (S)	0.016	0.062	0.25 (S)
OXA-48 ΔOmpK35/36	0.5 (S)	0.25 (S)	0.5	8 (I)	0.125	1	0.25 (S)

^*a*^ Susceptibilities are classified according to EUCAST clinical breakpoints, version 13.0. Clinical breakpoints are not available for meropenem–avibactam.

^*b*^ CAZ, ceftazidime; CAZ-AVI, ceftazidime–avibactam with the avibactam concentration fixed to 4 mg/L; CAZ-AVI_ratio_, ceftazidime–avibactam in a 4:1 ratio; COL, colistin; MEM, meropenem; MEM-AVI, meropenem–avibactam with the avibactam concentration fixed to 4 mg/L; MEM-AVI_ratio_ meropenem–avibactam in a 2:1 ratio.

### Concentration-dependent effects of avibactam

**Fig 1:**
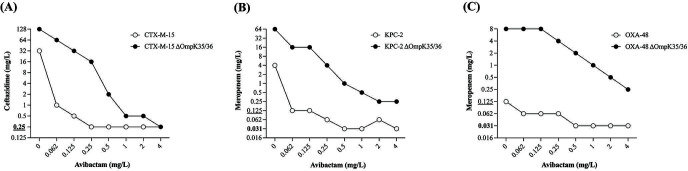
Concentration-dependent effect of avibactam against *Klebsiella pneumoniae* ATCC 35657 constructs. The lowest bactericidal β-lactam concentration for each avibactam concentration, as determined by spot assay, is plotted for (**A**) ceftazidime–avibactam against the CTX-M-15-producing strains, (**B**) meropenem–avibactam against the KPC-2-producing strains, and (**C**) meropenem–avibactam against the OXA-48-producing strains. For comparison, the lowest bactericidal concentration of the β-lactam against the parental strain is marked in bold on the y-axis, and the lowest bactericidal β-lactam concentration against the ΔOmpK35/36 strain is underlined.

Avibactam essentially restored the MICs of ceftazidime and meropenem in β-lactamase-producing strains (Table 1). However, there was sometimes a significant (4- to 16-fold) difference between the standard MIC and the MIC_ratio_. To determine the concentration-dependent effects of avibactam, we screened the individual activities of ceftazidime and meropenem in combination with a wide range of avibactam concentrations by time-lapse microscopy screening complemented with 24-h spot assay (Fig. 1; Fig. S1). The inhibition effect of avibactam was evaluated by determining the lowest avibactam concentrations required to restore the bactericidal activity of the β-lactam antibiotics (Fig. S2). For the CTX-M-15 construct, 0.25 mg/L of avibactam was sufficient to achieve the same antibacterial effects with ceftazidime compared with the parental strain, and 4 mg/L was required for the CTX-M-15 ΔOmpK35/36 strain (Fig. 1A; Fig. S1A). Similarly, 0.5 mg/L of avibactam was sufficient to achieve the same antibacterial effects with meropenem against the KPC-2 or OXA-48 constructs compared with the parental strain, whereas >4 mg/L of avibactam was needed to obtain a similar activity against the KPC-2 and OXA-48-producing strains with ΔOmpK35/36 (Fig. 1B and C; Fig. S1B and C).

### Screening of combination effects with BLBLIs and colistin

The activity of colistin and BLBLI combinations was evaluated by time-lapse microscopy screening with 24-h cell viability testing (spot assay). Fixed concentrations of 0.125 and 0.5 mg/L of avibactam were used based on the observed concentration-dependent effects of avibactam: 0.125 mg/L showed no inhibitory effects, and 0.5 mg/L rendered maximum β-lactamase inhibition against strains with intact porins. The automated background-corrected absorption (BCA) and maximum segmentation extracted surface area (SESA_max_) readouts showed significant discrepancies compared with spot assay results (Fig. S1 to S3). Specifically, the SESA_max_ cut-off was sometimes not attained despite viable counts >10^6^ CFU/mL at 24 h, most likely due to clumping of bacteria. In addition, bacterial filamentation may have generated unusually low SESA_max_ values and unusually high BCA values relative to the viable counts. Therefore, we decided to use only spot assay results in the analysis.

#### CTX-M-15 strains

**Fig 2:**
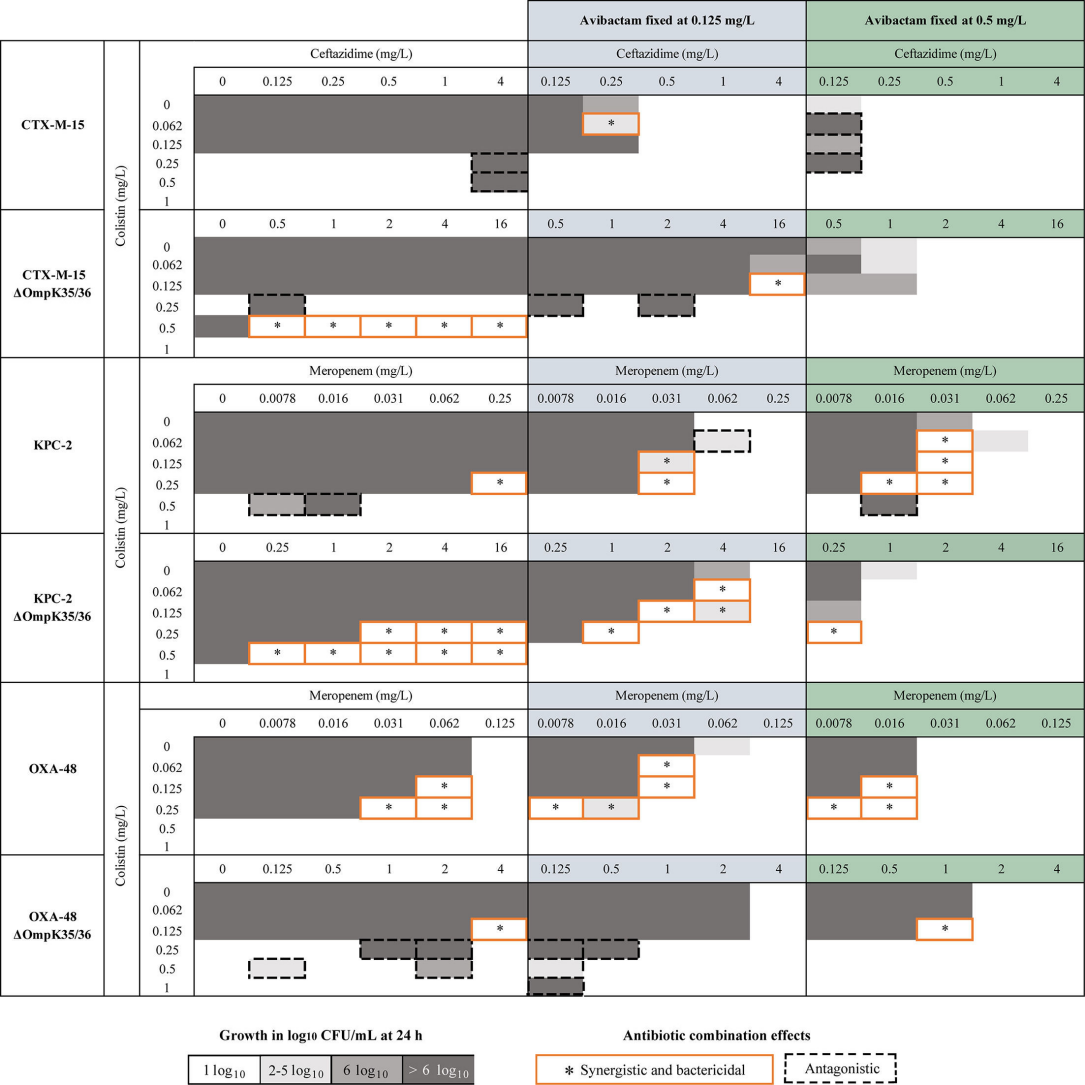
Screening for combination effects of colistin with ceftazidime or ceftazidime–avibactam against the CTX-M-15-producing *Klebsiella pneumoniae* strains and colistin combined with meropenem or meropenem–avibactam against the KPC-2- and OXA-48-producing strains. No visible growth was recorded as 1 log_10_ CFU/mL (white box).

Colistin combined with ceftazidime–avibactam showed synergy in one case against the CTX-M-15 strain (Fig. 2; Fig. S3A). Against the CTX-M-15 ΔOmpK35/36 strain, colistin showed synergy both in combination with ceftazidime (five cases) and ceftazidime–avibactam (one case) with the avibactam concentration fixed to 0.125 mg/L. No synergy was found when avibactam was added to 0.5 mg/L.

#### KPC-2 strains

Colistin and meropenem showed synergy against the KPC-2 strain at one of the tested concentration set-ups. The three-drug combination showed synergy (six cases), also at colistin concentrations below the MIC (<0.25 mg/L) (Fig. 2; Fig. S3B). Against the KPC-2 ΔOmpK35/36 strain, synergy was frequently found with colistin and meropenem (eight cases) and was also observed with the three-drug combinations (five cases), including colistin concentrations below the MIC. In some cases, the presence of avibactam reduced the amount of colistin needed to obtain synergy by 2- to 4-fold.

#### OXA-48 strains

Against the OXA-48 strain, synergy was observed with meropenem (three cases) and with meropenem–avibactam (seven cases), sometimes at colistin concentrations below the MIC (Fig. 2; Fig. S3C). Against the OXA-48 ΔOmpK35/36 strain, colistin demonstrated synergy in only one case with meropenem and meropenem–avibactam, respectively.

### Time-kill experiments

**Fig 3:**
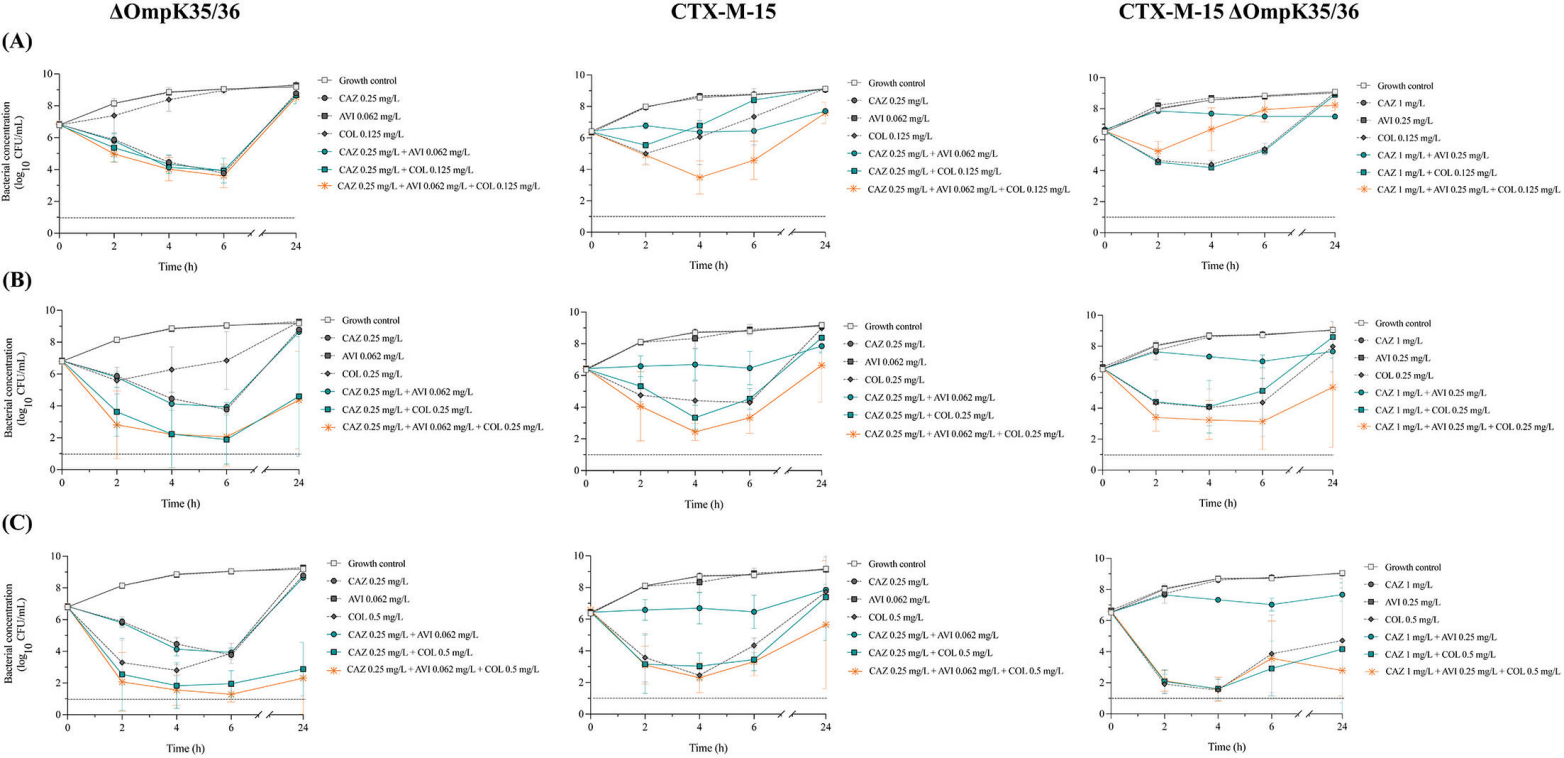
Time-kill experiments with *Klebsiella pneumoniae* ATCC 35657 constructs exposed to ceftazidime, avibactam, and colistin, alone and in combination; concentrations of ceftazidime–avibactam at 0.5× MIC_ratio_ and colistin at concentrations of (**A**) 0.5× MIC, (**B**) 1× MIC, and (**C**) 2× MIC. Mean bacterial concentrations and standard deviations (error bars) are presented. LOD (1 log_10_ CFU/mL) is marked with a dotted line. Abbreviations: AVI, avibactam; CAZ, ceftazidime; COL, colistin.

**Fig 4:**
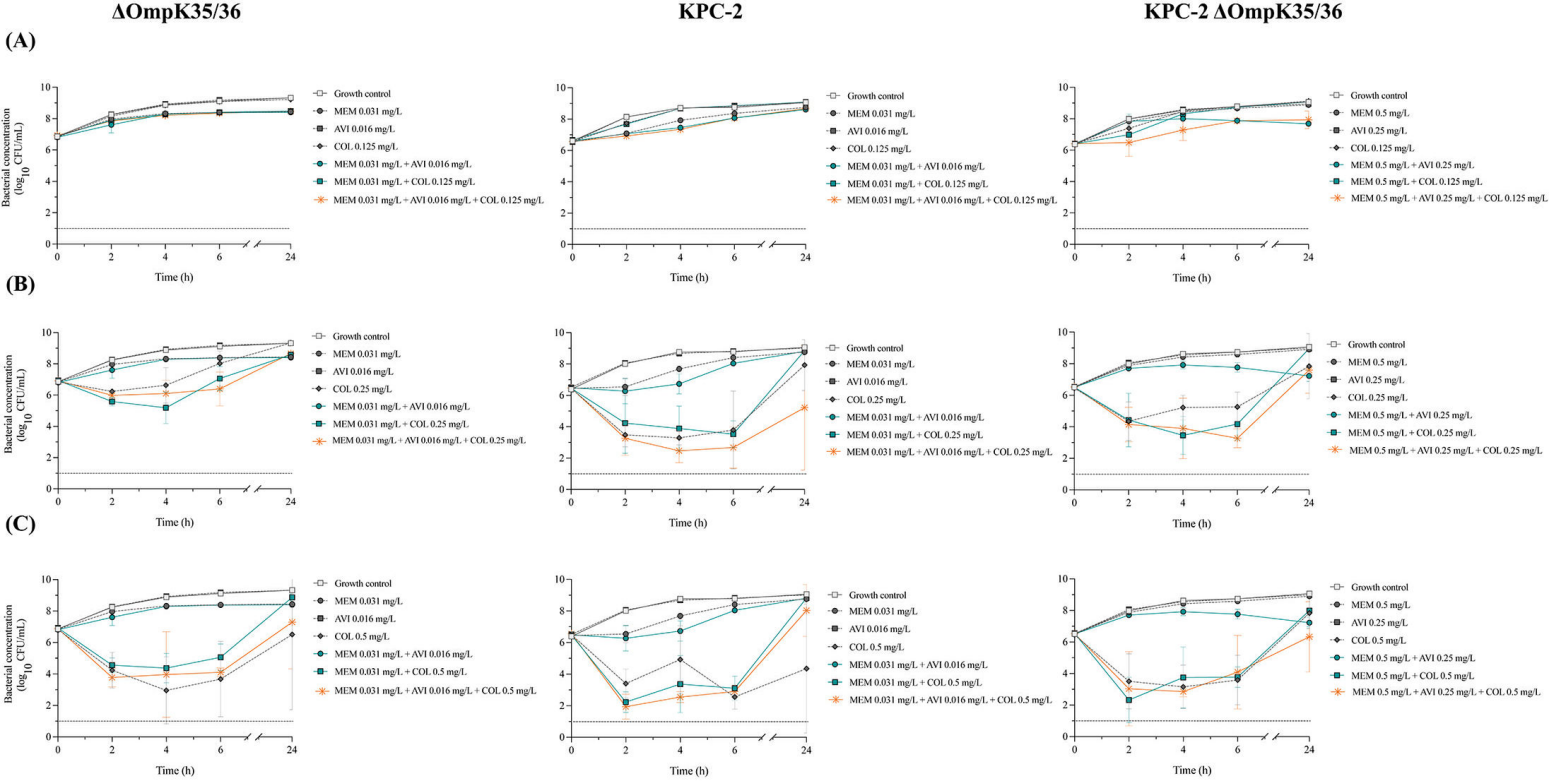
Time-kill experiments with *K. pneumoniae* ATCC 35657 constructs exposed to meropenem, avibactam, and colistin, alone and in combination; concentrations of meropenem–avibactam at 0.5× MIC_ratio_ and colistin at concentrations of (**A**) 0.5× MIC, (**B**) 1× MIC, and (**C**) 2× MIC. Mean bacterial concentrations and standard deviations (error bars) are presented. LOD (1 log_10_ CFU/mL) is marked with a dotted line. Abbreviations: AVI, avibactam; COL, colistin; MEM, meropenem.

**Fig 5:**
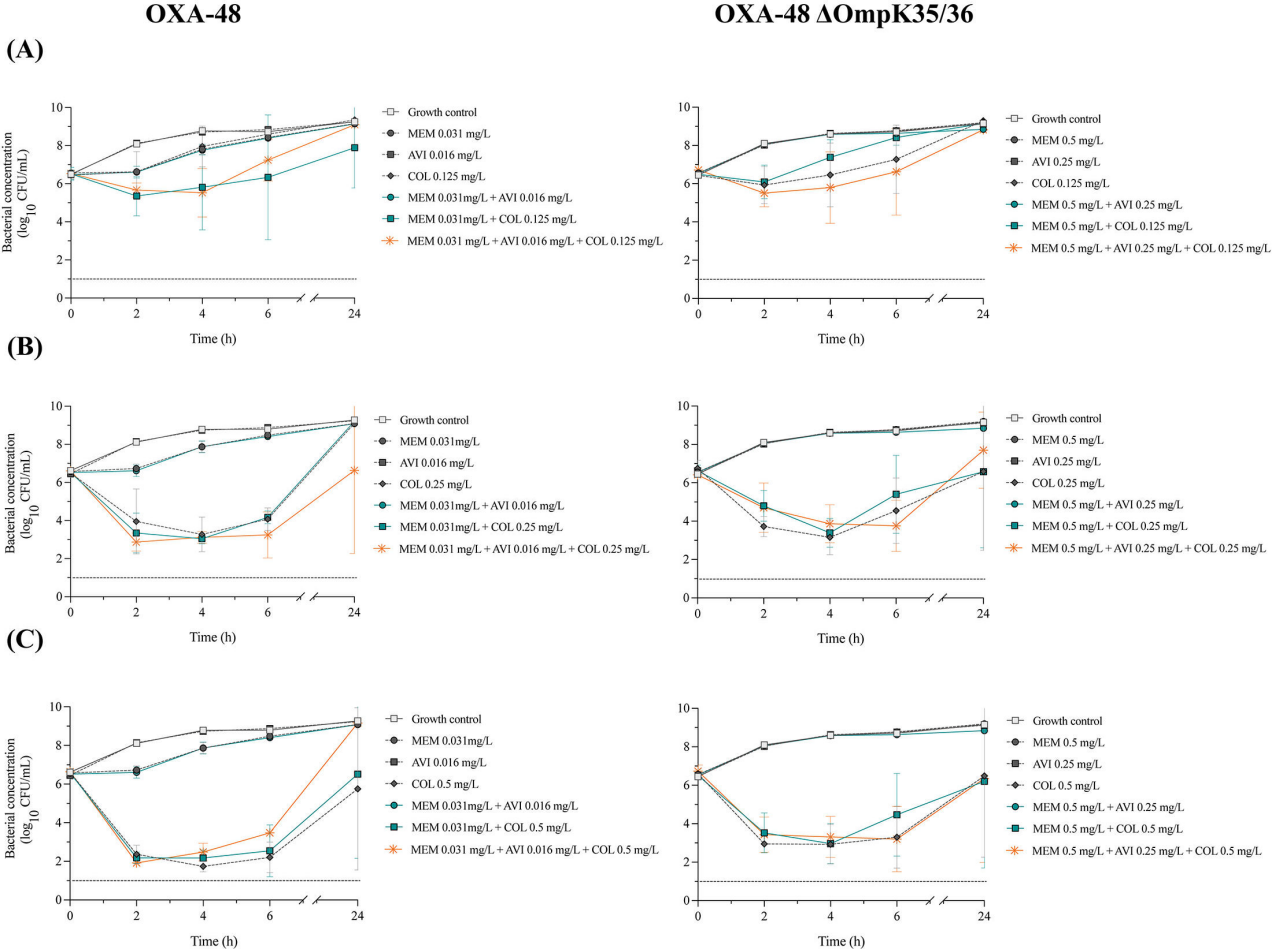
Time-kill experiments with *Klebsiella pneumoniae* ATCC 35657 constructs exposed to meropenem, avibactam, and colistin, alone and in combination; concentrations of meropenem–avibactam at 0.5× MIC_ratio_ and colistin at concentrations of (**A**) 0.5× MIC, (**B**) 1× MIC, and (**C**) 2× MIC. Mean bacterial concentrations and standard deviations (error bars) are presented. LOD (1 log_10_ CFU/mL) is marked with a dotted line. Abbreviations: AVI, avibactam; COL, colistin; MEM, meropenem.

In the time-kill experiments, we evaluated the activity of colistin and BLBLIs using concentration ratios of ceftazidime–avibactam (4:1) and meropenem–avibactam (2:1). Colistin alone at 0.5× MIC showed limited activity against all strains, and 1× or 2× MIC typically resulted in significant initial killing at 2–6 h followed by regrowth (Fig. 3 to 5).

#### ΔOmpK35/36 strain without β-lactamase production

Ceftazidime alone showed a bactericidal effect at 6 h followed by regrowth within 24 h (Fig. 3). The combination of ceftazidime and colistin at 1× MIC resulted in synergistic and bactericidal effects at 4 h and synergy at 24 h. With a higher colistin concentration of 2× MIC, the combination was synergistic and bactericidal at 24 h. In contrast, meropenem alone did not exhibit a significant antibacterial effect, and no synergy was detected in combination with colistin (Fig. 4). As expected, adding avibactam to either of the other agents did not significantly improve the antibacterial activity, as no β-lactamases were present.

#### CTX-M-15 strains

Ceftazidime–avibactam generally showed higher activity than ceftazidime alone (Fig. 3). Colistin at 0.5× MIC in combination with ceftazidime–avibactam demonstrated synergy at 4 h against the CTX-M-15 strain. Against the CTX-M-15 ΔOmpK35/36 strain, synergistic effects were observed at 24 h when colistin at 1× MIC was combined with ceftazidime–avibactam, whereas antagonism was observed at 4 and 6 h with colistin at 0.5× MIC. The three-drug combinations frequently showed bactericidal effects between 2 and 6 h, with the two higher colistin concentrations against both strains. Still, they did not show significantly better killing than colistin alone or colistin plus ceftazidime.

#### KPC-2 strains

For both KPC-2-producing strains, meropenem and meropenem–avibactam demonstrated limited antibacterial activity at the tested concentrations (Fig. 4). The three-drug combination often showed bactericidal effects between 2 and 6 h when using the higher colistin concentrations but generally resulted in similar killing as colistin alone or combined with meropenem. Against the strain with intact porins, colistin at 1× MIC in combination with meropenem–avibactam resulted in synergy at 24 h, whereas colistin at 2× MIC combined with meropenem or meropenem–avibactam resulted in antagonism at 24 h.

#### OXA-48 strains

As for the KPC-2 strains, limited effects were observed for meropenem and meropenem–avibactam against both OXA-48-producing strains (Fig. 5). The three-drug combinations showed bactericidal effects between 2 and 6 h when using the higher colistin concentrations. However, bacterial killing was typically similar to that of colistin alone or in combination with meropenem. Against the OXA-48 strain with intact porins, synergy was found with colistin at 0.5× MIC in combination with meropenem at 4 and 6 h, and with colistin at 1× MIC in combination with meropenem–avibactam at 24 h. However, antagonism was seen at 24 h with colistin at 2× MIC in combination with meropenem–avibactam. No synergistic effects were detected against the OXA-48 ΔOmpK35/36 strain.

## DISCUSSION

This study evaluated the impact of porin loss on the synergistic potential of colistin in combination with BLBLIs against β-lactamase-producing *K. pneumoniae* with or without the major porins OmpK35/36. Because synergy with colistin is assumed to be mediated by membrane disruption, we hypothesized that synergy would be observed more frequently against strains without porin expression. To test this hypothesis, we constructed *K. pneumoniae* strains producing CTX-M-15, KPC-2, or OXA-48 with the major porin-encoding genes (*ompK35*, *ompK36*) either intact or knocked out. Avibactam was selected as the enzyme inhibitor as it is active against commonly encountered β-lactamases in MDR *K. pneumoniae*, including CTX-M-15, KPC-2, and OXA-48. CTX-M-15 hydrolyzes ceftazidime efficiently but meropenem poorly (24). In contrast, KPC-2 and OXA-48 hydrolyze ceftazidime poorly but meropenem more efficiently (25, 26). Hence, avibactam was paired with ceftazidime against the CTX-M-15-producing strains and with meropenem against the KPC-2- and OXA-48-producing strains.

Antibiotic susceptibility testing confirmed the MICs of the constructed strains to be in line with the presumed β-lactamase activities against ceftazidime and meropenem. Loss of porins OmpK35/36 alone had a limited impact on β-lactam susceptibility in the absence of β-lactamases. Yet, significant MIC elevations were noted for the β-lactams and BLBLIs when porin loss was combined with β-lactamase production.

The MICs of the constructed strains indicate that the loss of OmpK35/36 does not entirely prevent the entry of ceftazidime or meropenem into the cell. However, as previously reported, it may reduce the periplasmic drug concentration to a level where β-lactamases with low hydrolytic activity may cause significantly reduced susceptibility (9, 11, 12, 27, 28). For example, we found that the introduction of OXA-48 into the parental strain resulted in a mere 2-fold increase in meropenem MIC, yet in a porin-deficient background, the increase was an additional 128-fold. Furthermore, despite weak meropenem hydrolysis, CTX-M-15 in a porin-deficient background resulted in a 64-fold increase in meropenem MIC. This observation illustrates the impact of combined resistance mechanisms, which is a common feature of clinical MDR Gram-negative isolates.

An apparent dose-dependent effect of avibactam was observed. In β-lactamase-producing strains with intact porins, low avibactam concentrations had an immediate impact on β-lactam susceptibility. In contrast, a more gradual effect was observed in strains without porins, where 4- to 16-fold higher avibactam concentrations were required to inhibit the β-lactamase activity. These findings illustrate that OmpK35 and OmpK36 play a central role in the entry of avibactam. However, avibactam may also diffuse through other porins (e.g., OmpK37 and PhoE) in the event of OmpK35 and OmpK36 deficiencies (11, 14).

In the screening, the pattern of combination effects varied across the strains and tested BLBLIs, with synergistic and bactericidal effects most frequently found against the KPC-2-producing isolates. Colistin, ceftazidime, and avibactam combined demonstrated synergy only at one of the tested concentration set-ups against each CTX-M-15-producing strain, regardless of porin expression. For the KPC-2-producing strain with intact porins, synergistic effects were found at several concentrations with colistin in combination with meropenem or meropenem–avibactam. Yet, synergy generally ranged over a larger concentration span in the absence of porins. However, this was not the case for the OXA-48-producing strains without porins, where synergy was only observed at a few of the tested concentrations set-ups.

Overall, synergy was less commonly observed in the time-kill experiments and no consistent difference in synergistic effects could be seen between strains with or without porins. Although the three-drug combinations were often bactericidal with the higher colistin concentrations (1× or 2× MIC), the effect was generally not significantly better than colistin alone or colistin plus ceftazidime or meropenem. The difference in results between methods may be partly due to differences in growth conditions and the higher total bacterial amount in the time-kill experiments (due to a larger working volume), which increases the risk of inoculum effects and resistance development. Also, the effect could be concentration-dependent. Although a range of fixed drug concentrations was used in the screening, BLBLIs were only tested at concentrations of 0.5× MIC_ratio_ in the time-kill experiments.

The standard method for susceptibility testing of BLBLIs per CLSI and EUCAST recommendations is broth microdilution using a fixed concentration of the β-lactamase inhibitor (29–31). Hence, most *in vitro* studies with avibactam, including time-kill experiments evaluating polymyxins in combination with ceftazidime–avibactam against KPC-producing *K. pneumoniae,* have added avibactam to a fixed concentration of 4 mg/L (20–23, 32, 33). However, considering that BLBLIs are administered in ratios (e.g., 4:1 for ceftazidime–avibactam) in clinical practice, we employed ratios in the experiments to better reflect the concentration relationship between the drugs in treated patients. Also, given that the objective was to explore the influence of colistin on β-lactam and avibactam uptake, this approach had the additional advantage of avoiding excessive amounts of the β-lactamase inhibitor. Consequently, the avibactam concentrations in our experiments were lower than in most previous studies (the avibactam concentration at the MIC_ratio_ was consistently <4 mg/L), which may have negatively affected the probability of finding synergy.

Although the screening was conducted using time-lapse microscopy, we decided to use only the results of the spot assay in the analysis of combination effects. The problems with the automated readout based on BCA and SESA_max_ were partly attributed to the sticky phenotype of *K. pneumoniae* ATCC 35657, resulting in cell clustering, which challenged the identification of individual bacteria. Also, filamentation during exposure to ceftazidime and BLBLIs, a phenomenon previously reported with aztreonam against *Enterobacterales* (34, 35), was falsely interpreted as high bacterial concentrations. Refinement of the growth kinetic algorithms of the oCelloScope is required for antibiotics that induce filamentation.

The systematic approach to assessing the synergistic potential of colistin in combination with BLBLIs against strains with different β-lactamases, with or without porins, in a well-defined genetic context is a strength of this study. Chromosomal insertion of β-lactamase genes was suitable for this purpose. However, we recognize that clinical MDR Gram-negative bacteria usually carry β-lactamase genes on plasmids, and often in high copy numbers (1, 36). High variability in results was noted for colistin alone, complicating the analysis of combination effects. Such variability is often observed with polymyxins and is likely due to biological variation as well as resistance development (37–40). We only conducted one replicate in the screening but tested several drug concentrations and, in addition, performed time-kill experiments to provide more detailed information on combination effects. Moreover, to some extent, combination effects depend on the selected antibiotic concentrations. Studies using a more extensive set of strains, including clinical isolates that often carry multiple resistance mechanisms, are warranted to validate our findings.

In conclusion, synergistic effects were observed with colistin combined with ceftazidime–avibactam or meropenem–avibactam against β-lactamase-producing *K. pneumoniae* in screening experiments. Positive combination effects were less pronounced in the time-kill experiments, and synergy was rarely detected. No clear association between porin loss and combination effects could be demonstrated, indicating that the presence or absence of porins alone does not determine synergistic interactions and cannot be used as a predictor of synergy. However, our findings do not preclude a potential clinical benefit of colistin in combination with BLBLIs. More research is warranted to increase our understanding of the bacterial genetic determinants of combination effects and the mechanisms of synergy.

## MATERIALS AND METHODS

### Growth conditions and antibiotics

Cation-adjusted Mueller–Hinton (MH-II) (BD Diagnostics, Sparks, MD, USA) broth or agar was used unless otherwise specified. Viable counts were read after 24 h of incubation at 37°C. Antibiotics (colistin, C4461; meropenem, PHR1772; ceftazidime, C3809; avibactam, A169351) were purchased from Sigma-Aldrich (Merck KGaA, Darmstadt, Germany) and prepared according to the manufacturer’s instructions.

### Strain construction

Strain construction was performed in *K. pneumoniae* ATCC 35657 (NZ_CP015134) using λ-red recombineering with the pSIM5-*tet* plasmid as previously described (41). In short, a *kan-sacB* cassette was introduced chromosomally into *galK*. Transformants were recovered overnight in SOC (Super Optimal broth with Catabolite repression) medium and selected on 50 mg/L kanamycin. The *kan-sacB* cassette was subsequently exchanged for different β-lactamase genes with their native promoter sequences and flanked by transcriptional terminators. The *bla*_KPC-2_ and *bla*_OXA-48_ genes with flanking transcriptional terminators were amplified from *Escherichia coli* ATCC 25922 constructs used in a previous study (41). The *bla*_CTX-M-15_ gene was amplified from a plasmid (pUUH239.2) originally isolated from an MDR clinical *K. pneumoniae* strain (42, 43). The porin genes were knocked out by exchanging *ompK35* and *ompK36* with the *kan-sacB* cassette, followed by removal of the cassette using oligonucleotides with homologies upstream and downstream of the cassettes. Primers are presented in Table S1. Insertions were verified with PCR, local Sanger sequencing (Eurofins Genomics, Aarhus, Denmark), and whole genome sequencing (WGS).

### Whole genome sequencing

WGS was performed using Illumina MiSeq (Illumina Inc., San Diego, CA, USA). Genomic DNA was prepared using the Epicentre MasterPure DNA purification Kit (Illumina Inc.) according to the manufacturer’s instructions. Sequences were assembled against the reference *K. pneumoniae* ATCC 35657 (NZ_CP015134) in CLC Genomics Workbench version 23 (CLCbio, Qiagen) and analyzed for genetic variations (SNPs, InDels) in CLC Main Workbench version 23 (CLCbio, Qiagen).

### Antibiotic susceptibility testing

Susceptibility testing was performed by broth microdilution according to EUCAST recommendations (29, 44–46). For the BLBLIs, two different MICs were determined. First, the MIC was determined with a fixed avibactam concentration of 4 mg/L according to EUCAST recommendations (29). Second, the MIC was tested at fixed ratios of ceftazidime–avibactam and meropenem–avibactam, referred to as MIC_ratio_. Ceftazidime–avibactam is available for clinical use at a 4:1 ratio (2 g:0.5 g), which was therefore used in the MIC_ratio_ determination. A 2:1 ratio was selected for meropenem–avibactam based on the standard doses of meropenem (1 g) and avibactam (0.5 g) (29). MIC determinations were conducted in at least two biological replicates. If the results differed, a third replicate was performed, and the median value was used.

### Screening by time-lapse microscopy and cell viability testing (spot assay)

Evaluation of concentration-dependent effects of avibactam on β-lactam susceptibility and screening of the activity of single drugs and combinations were performed using automated time-lapse microscopy (oCelloScope, Philips BioCell A/S, Allerød, Denmark) as previously described (47, 48). The experiments were performed in 96-well microtiter plates (Greiner Bio-One GmbH, Frickenhausen, Germany) with antibiotics added to starting bacterial inocula of ~10^6^ CFU/mL in an exponential growth phase. The oCelloScope instrument was kept at 37°C, and images were generated every 7.5 min for 24 h. Background-corrected absorption (BCA) >8.0 and maximum segmentation extracted surface area (SESA_max_) >5.8, calculated using the UniExplorer software version 11.0 (Philips BioCell A/S, Allerød, Denmark), were used as cut-off values to indicate bacterial growth >10^6^ CFU/mL at 24 h (34, 35). *E. coli* ATCC 25922 was used as the quality control strain for ceftazidime, meropenem, and colistin, and *K. pneumoniae* ATCC 700603 was used as the quality control strain for avibactam. Following the 24-h experiments, 10 µL of undiluted and serially diluted samples from the wells was spotted onto plates for viable counts. The screening experiments were performed in one replicate. The lower limit of detection (LOD) in the spot assay was 2 log_10_ CFU/mL, and no visible growth was noted as 1 log_10_ CFU/mL.

First, the concentration-dependent effect of avibactam was determined by combining ceftazidime or meropenem with increasing avibactam concentrations ranging from 0.062 to 4 mg/L against the β-lactamase-producing strains with and without OmpK35/36. Second, screening for antibacterial effects was performed with ceftazidime, avibactam, and colistin alone and in two- and three-drug combinations against the CTX-M-15 and the CTX-M-15 ΔOmpK35/36 strains, and with meropenem, avibactam, and colistin alone and in combinations against the KPC-2, KPC-2 ΔOmpK35/36, OXA-48, and OXA-48 ΔOmpK35/36 strains. Antibiotic concentrations close to the MIC_ratio_ were used to increase the likelihood of detecting combination effects. Avibactam was added in fixed concentrations of 0.125 and 0.5 mg/L in the screening experiments.

### Time-kill experiments

Static time-kill experiments were performed at 37°C using a starting inoculum of ~10^6^ CFU/mL in the exponential growth phase. Samples for viable counts were collected at 0, 2, 4, 6, and 24 h. All experiments were performed in at least duplicates. The LOD was 1 log_10_ CFU/mL, and no visible growth was noted at 1 log_10_ CFU/mL. The activities of colistin and ceftazidime–avibactam (4:1 ratio) alone or in combination were tested against the CTX-M-15, CTX-M-15 ΔOmpK35/36, and ΔOmpK35/36 strains. Colistin and meropenem–avibactam (2:1 ratio) regimens were tested against the KPC-2, KPC-2 ΔOmpK35/36, OXA-48, OXA-48 ΔOmpK35/36, and ΔOmpK35/36 strains. Based on preliminary time-kill data with colistin and BLBLIs alone (Fig. S4), we decided to use colistin concentrations of 0.5×, 1×, and 2× MIC. BLBLI concentrations of 0.5× MIC_ratio_ were selected to enable the detection of potential synergistic effects with the three-drug combinations and avoid extensive bacterial killing by the BLBLIs alone.

### Definitions of synergy, antagonism, and bactericidal effect

Mean bacterial concentrations were used in the time-kill analyses. No visible growth was noted as 1 log_10_ CFU/mL so as not to overestimate the effect. Synergy was defined as a ≥2 log_10_ decrease in CFU/mL with the combination compared to the most effective single antibiotic, and antagonism as a ≥2 log_10_ CFU/mL increase with the combination. The most effective single-drug or two-drug combination was used as a comparator to the three-drug combinations. A bactericidal effect was defined as a ≥3 log_10_ reduction in CFU/mL compared with the starting inoculum (49).

## Data Availability

Raw sequencing data have been deposited in the NCBI Sequence Read Archive under BioProject accession number PRJNA1128528.

## SUPPLEMENTAL MATERIAL

Table S1; Figures S1 to S4 aac.00762-24-s0001.pdf Primers used in the study, raw data from the time-lapse microscopy screening, and time-kill curves.

ASM does not own the copyrights to Supplemental Material that may be linked to, or accessed through, an article. The authors have granted ASM a non-exclusive, world-wide license to publish the Supplemental Material files. Please contact the corresponding author directly for reuse.
