# Supplementary material for: Impact of porin deficiency on the synergistic potential of colistin in combination with β-lactam/β-lactamase inhibitors against ESBL- and carbapenemase-producing Klebsiella pneumoniae
Source: Antimicrob Agents Chemother. 2024 Oct 4;68(11):e00762-24. doi: 10.1128/aac.00762-24 (PMC11539213; doi:10.1128/aac.00762-24)
Supplement: Table S1; Figures S1 to S4 — Primers used in the study, raw data from the time-lapse microscopy screening, and time-kill curves. [file aac.00762-24-s0001.pdf]

Supplemental Material

Impact of porin deficiency on the synergistic potential of colistin in combination with β-lactam/β-lactamase inhibitors against ESBL- and carbapenemase-producing *Klebsiella pneumoniae*

Lisa Allander, Karin Vickberg, Elin Fermér, Thomas Söderhäll, Linus Sandegren, Pernilla Lagerbäck, Thomas Tängdén.

Table S1. Primers used in the study. Gradient PCR (Thermo Scientific™ Phusion™ High-Fidelity DNA Polymerase) was used for amplification.

Application	Sequence (5´→3´)
Amplification of *kan-sacB* cassette with homology to ATCC 35657 *ompK35*	Forward: TCCTGCTTTGAAGGCATATCACGAAGGGGTGTACTGCAGATGTAGGCTGGAGCTGCTTC<br>Reverse: CACGTGTTCATATAAAAAATATTAATGAGGGTAATAAATACATATGAATATCCTCCTTAGTTCC
Amplification of *kan-sacB* cassette with homology to ATCC 35657 *ompK36*	Forward: AAAGGCGCCAGAGGCGCCTTTTTGTTATGCAGCTTGCAACTGTAGGCTGGAGCTGCTTC<br>Reverse: GGCATAATAAAAGGCATATAACAAACAGAGGGTTAATAACCATATGAATATCCTCCTTAGTTCC
Amplification of *kan-sacB* with homologies to ATCC 35657 *galk*	Forward: GCGCCGTCAGCGACGTCCATTTTCGTGAATCCGGAGTGTATGTAGGCTGGAGCTGCTTC<br>Reverse: GCTGACCGTCCGGGGCCAGCGCGGTGGTTTGCGTTAGCATTCATATGAATATCCTCCTTAGTTCC
Amplification of *cat-sacB* cassette with regions homologous to ATCC 25922 *bgl*	Forward: GCTCGATAAACTGCTGGCAGAAAAAGATAGCGATAAATAATTCACCAGACAAATCCCAAT<br>Reverse: GACTGTTCTGAATGCGACGATATTTAAGGTGCTTTATTGGAATATCCCTTTATGGTGCAAAG
ompK35 ATCC 35657 oligonucleotide	TCCTGCTTTGAAGGCATATCACGAAGGGGTGTACTGCAGATATTTATTACCCTCATTAATATTTTTTATATGAACACGTG
ompK36 ATCC 35657 oligonucleotide	AAAGGCGCCAGAGGCGCCTTTTTGTTATGCAGCTTGCAACGTTATTAACCCTCTGTTTGTTATATGCCTTTTATTATGCC
Amplification of pUUH *bla*$_{\text{CTX-M-15}}$	Forward: AAGATGAAATCAATGATTTA<br>Reverse: TCTAAGGCGATAAACAAAAA
Amplification of *bla*$_{\text{CTX-M-15}}$ with homologies to ATCC 25922 *cat-sacB*	Forward: AGCCGGATTAATAATCTGGCTTTTTATATTCTCTGTCGACAAGATGAAATCAATGATTTA<br>Reverse: AAAGCCCCGAGCGGTTAAACTCAGGGCTTTATTTGAGCTCTCTAAGGCGATAAACAAAAA
Amplification of β-lactamase gene with homologies to insertion site in ATCC 35657 *kan-sacB*	Forward: GCGCCGTCAGCGACGTCCATTTTCGTGAATCCGGAGTGTATTCACCAGACAAATCCCAAT<br>Reverse: GCTGACCGTCCGGGGCCAGCGCGGTGGTTTGCGTTAGCATATATCCCTTTATGGTGCAAAG
Screening ATCC 35657 *ompK35*	Forward: TACAGAAAAGCAGGACCGAA<br>Reverse: AGAACTTATTGACGGCAGTG
Screening ATCC 35657 *ompK36*	Forward: CGACAAGAGTATACCAGCGA<br>Reverse: GCCGACTGATTAGAAGGGTA
Screening insertion site ATCC 25922 *bgl*	Forward: CGCTGCCAGAATATTTGTGA<br>Reverse: CGCCTTTTCTAATAGCTCAA
Screening insertion site ATCC 35657 *galk*	Forward: CTTATTGCGCAGGGTCACCT<br>Reverse: CGAAACTCAGCGCGATCTGA

(A)

CTX-M-15 / Time-lapse microscopy	0		CAZ 0.062		CAZ 0.125		CAZ 0.25		CAZ 0.5		CAZ 1		CAZ 2		CAZ 4		CAZ 8		CAZ 16		CAZ 32		CAZ 64	
	$SESA_{max}$	BCA	$SESA_{max}$	BCA	$SESA_{max}$	BCA	$SESA_{max}$	BCA	$SESA_{max}$	BCA	$SESA_{max}$	BCA	$SESA_{max}$	BCA	$SESA_{max}$	BCA	$SESA_{max}$	BCA	$SESA_{max}$	BCA	$SESA_{max}$	BCA	$SESA_{max}$	BCA
0	6.0	9.0	5.9	9.0	6.0	9.0	5.9	9.0	6.1	9.0	6.0	9.0	6.0	9.0	6.0	9.0	6.1	9.0	6.3	9.0	6.2	8.3	6.1	7.8
AVI 0.062	5.8	9.0	6.1	9.0	6.1	9.0	6.3	8.9	6.3	8.8	6.2	8.0	6.2	7.8	6.2	7.8	6.2	7.8	5.9	7.1	6.0	7.4	5.6	6.7
AVI 0.125	6.0	9.0	6.0	9.0	6.2	8.9	6.3	8.9	6.2	8.2	6.2	7.9	6.2	8.0	6.0	7.4	6.2	7.9	6.1	7.8	5.1	7.2	5.0	6.8
AVI 0.25	6.1	9.0	6.1	9.0	6.2	8.9	6.3	8.8	6.1	8.3	6.1	8.1	6.1	8.1	6.2	8.0	6.1	7.9	5.9	8.0	5.2	8.6	4.6	8.8
AVI 0.5	6.1	9.0	6.1	9.0	6.3	8.9	6.2	8.6	6.1	8.5	6.1	8.3	6.2	8.3	6.0	7.9	5.9	8.0	5.7	8.1	5.0	8.6	4.4	8.7
AVI 1	6.1	9.0	6.1	9.0	6.3	8.9	6.2	8.4	6.1	8.3	6.0	8.2	6.2	8.2	5.9	8.1	5.8	8.3	5.0	8.6	4.8	8.7	4.5	8.7
AVI 2	5.9	9.0	6.2	8.9	6.4	8.8	6.2	8.5	6.2	8.3	6.2	8.3	6.2	8.4	5.5	8.4	5.7	8.6	5.1	8.7	4.9	8.3	4.6	6.2
AVI 4	6.1	9.0	6.3	8.9	6.3	8.7	6.1	8.4	6.1	8.5	6.1	8.6	5.9	8.7	5.7	8.7	5.5	8.7	4.7	8.7	4.5	7.5	4.6	6.1

CTX-M-15 / Viable count (log_{10} CFU/mL)	0	CAZ 0.062	CAZ 0.125	CAZ 0.25	CAZ 0.5	CAZ 1	CAZ 2	CAZ 4	CAZ 8	CAZ 16	CAZ 32	CAZ 64
0	8	8	8	9	7	7	8	8	7	8	1	1
AVI 0.062	9	8	8	6	4	1	1	1	1	1	1	1
AVI 0.125	9	8	8	6	1	1	1	1	1	1	1	1
AVI 0.25	9	8	7	3	1	1	1	1	1	1	1	1
AVI 0.5	8	8	6	1	1	1	1	1	1	1	1	1
AVI 1	9	7	5	1	1	1	1	1	1	1	1	1
AVI 2	8	7	5	1	1	1	1	1	1	1	1	1
AVI 4	8	7	5	1	1	1	1	1	1	1	1	1

CTX-M-15 ΔOmpK35/36 / Time-lapse microscopy	0		CAZ 0.125		CAZ 0.25		CAZ 0.5		CAZ 1		CAZ 2		CAZ 4		CAZ 8		CAZ 16		CAZ 32		CAZ 64		CAZ 128	
	$SESA_{max}$	BCA	$SESA_{max}$	BCA	$SESA_{max}$	BCA	$SESA_{max}$	BCA	$SESA_{max}$	BCA	$SESA_{max}$	BCA	$SESA_{max}$	BCA	$SESA_{max}$	BCA	$SESA_{max}$	BCA	$SESA_{max}$	BCA	$SESA_{max}$	BCA	$SESA_{max}$	BCA
0	5.5	9.0	5.4	9.0	6.0	9.0	5.9	9.0	5.8	9.0	5.8	9.0	5.9	9.0	6.0	9.0	6.3	9.0	6.3	9.0	6.3	8.9	5.7	6.8
AVI 0.062	5.1	9.0	5.6	9.0	5.9	9.0	5.7	9.0	5.9	9.0	5.8	9.0	5.9	9.0	6.2	9.0	6.3	8.9	6.4	8.9	5.7	7.0	5.7	6.2
AVI 0.125	5.9	9.0	5.9	9.0	6.0	9.0	5.8	9.0	6.0	9.0	6.0	9.0	6.2	9.0	6.3	8.9	6.3	8.8	5.9	7.0	5.7	6.5	5.6	6.6
AVI 0.25	5.9	9.0	6.0	9.0	6.1	9.0	6.2	9.0	6.3	8.9	6.3	8.9	6.3	8.8	6.3	8.6	5.9	7.5	5.8	6.9	5.6	6.3	5.5	6.7
AVI 0.5	5.7	9.0	5.7	9.0	6.3	8.9	6.3	8.6	6.4	8.7	6.1	7.7	5.8	7.2	6.0	7.3	5.5	6.4	5.1	5.7	4.9	6.3	4.7	6.8
AVI 1	5.7	9.0	6.0	9.0	6.3	8.8	6.2	7.8	5.9	7.3	5.9	7.3	5.7	6.9	5.7	7.0	5.4	6.9	4.8	5.9	4.7	7.0	4.8	6.7
AVI 2	5.9	9.0	6.3	9.0	6.3	8.7	6.1	7.8	6.0	7.1	5.5	7.3	5.5	6.7	5.5	7.0	5.2	7.4	4.7	6.7	4.8	7.1	4.8	6.1
AVI 4	6.2	9.0	6.3	8.9	6.4	8.6	5.8	7.5	5.7	7.1	5.6	6.3	5.4	6.3	4.9	5.9	4.6	6.0	4.5	6.2	4.7	6.4	4.4	6.4

CTX-M-15 ΔOmpK35/36 / Viable count (log_{10} CFU/mL)	0	CAZ 0.125	CAZ 0.25	CAZ 0.5	CAZ 1	CAZ 2	CAZ 4	CAZ 8	CAZ 16	CAZ 32	CAZ 64	CAZ 128
0	9	9	9	9	7	8	8	8	8	8	6	2
AVI 0.062	9	9	8	8	8	8	8	7	7	6	1	1
AVI 0.125	9	8	8	8	8	7	7	7	6	1	1	1
AVI 0.25	9	8	7	7	7	6	6	5	3	1	1	1
AVI 0.5	9	8	7	4	4	1	1	1	1	1	1	1
AVI 1	8	7	6	1	1	1	1	1	1	1	1	1
AVI 2	8	7	4	1	1	1	1	1	1	1	1	1
AVI 4	7	6	2	1	1	1	1	1	1	1	1	1

Abbreviations: AVI, avibactam; CAZ, ceftazidime.

Figure S1. Automated time-lapse microscopy readouts and viable counts (log_{10} CFU/mL) with ceftazidime-avibactam and meropenem-avibactam (mg/L) against *K. pneumoniae* ATCC 35657 constructed strains **(A)** CTX-M-15 and CTX-M-15 ΔOmpK35/36 strains, **(B)** KPC-2 and KPC-2 ΔOmpK35/36 strains, **(C)** OXA-48 and OXA-48 ΔOmpK35/36 strains. The 24-hour BCA and $SESA_{max}$ values are presented, and values above the predefined cut-offs (BCA > 8.0, $SESA_{max}$ > 5.8) are marked in grey. For viable counts, no visible growth is recorded as 1 log_{10} CFU/mL. Bacterial densities > 6 log_{10} CFU/mL are marked in grey.

(B)

KPC-2 — Time-lapse microscopy

AVI	0 SESA$_{max}$	0 BCA	MEM 0.016 SESA$_{max}$	MEM 0.016 BCA	MEM 0.031 SESA$_{max}$	MEM 0.031 BCA	MEM 0.062 SESA$_{max}$	MEM 0.062 BCA	MEM 0.125 SESA$_{max}$	MEM 0.125 BCA	MEM 0.25 SESA$_{max}$	MEM 0.25 BCA	MEM 0.5 SESA$_{max}$	MEM 0.5 BCA	MEM 1 SESA$_{max}$	MEM 1 BCA	MEM 2 SESA$_{max}$	MEM 2 BCA	MEM 4 SESA$_{max}$	MEM 4 BCA	MEM 8 SESA$_{max}$	MEM 8 BCA
0	5.8	9.0	6.3	8.9	6.4	8.9	6.3	8.9	6.2	9.0	6.2	9.0	6.1	9.0	6.1	9.0	6.1	9.0	4.0	7.7	4.6	7.4
AVI 0.062	5.9	9.0	6.3	8.9	6.3	8.9	6.3	8.9	5.9	7.9	5.6	7.6	5.6	7.2	5.1	7.0	4.6	6.2	4.6	8.3	4.7	6.7
AVI 0.125	6.0	9.0	6.3	8.9	6.3	8.9	6.4	9.0	6.0	7.9	5.8	7.8	5.6	7.7	5.0	7.5	5.0	7.6	4.5	7.6	4.5	7.3
AVI 0.25	5.4	9.0	6.3	8.9	6.3	8.9	6.1	8.3	5.9	8.1	5.8	7.9	5.4	7.7	5.0	7.7	5.1	8.1	4.5	8.2	4.4	7.5
AVI 0.5	6.3	8.8	6.4	8.9	6.3	8.5	6.1	8.3	6.0	8.2	5.6	7.9	5.7	8.0	5.1	7.7	4.6	8.3	4.3	8.1	4.6	6.7
AVI 1	6.2	8.9	6.3	8.8	6.3	8.5	6.1	8.5	6.0	8.4	5.7	8.3	5.6	8.1	4.8	7.7	4.7	8.5	4.8	8.0	4.5	8.8
AVI 2	6.1	9.0	6.4	8.8	6.2	8.1	5.7	7.5	5.4	6.6	ND		ND		ND		ND		ND		ND	
AVI 4	6.2	8.9	6.4	8.8	6.1	7.9	5.7	7.2	5.7	7.1	ND		ND		ND		ND		ND		ND	

KPC-2 — Viable count (log_{10} CFU/mL)

AVI	0	MEM 0.016	MEM 0.031	MEM 0.062	MEM 0.125	MEM 0.25	MEM 0.5	MEM 1	MEM 2	MEM 4	MEM 8
0	9	7	8	8	8	8	8	8	7	1	1
AVI 0.062	8	8	8	7	1	1	2	1	1	1	1
AVI 0.125	9	7	7	7	1	1	1	1	1	1	1
AVI 0.25	8	7	6	1	1	1	1	1	1	1	1
AVI 0.5	7	7	1	1	1	1	1	1	1	1	1
AVI 1	8	7	3	1	1	1	1	1	1	1	1
AVI 2	7	6	6	1	1	ND	ND	ND	ND	ND	ND
AVI 4	7	7	1	1	1	ND	ND	ND	ND	ND	ND

KPC-2 ΔOmpK35/36 — Time-lapse microscopy

AVI	0 SESA$_{max}$	0 BCA	MEM 0.062 SESA$_{max}$	MEM 0.062 BCA	MEM 0.125 SESA$_{max}$	MEM 0.125 BCA	MEM 0.25 SESA$_{max}$	MEM 0.25 BCA	MEM 0.5 SESA$_{max}$	MEM 0.5 BCA	MEM 1 SESA$_{max}$	MEM 1 BCA	MEM 2 SESA$_{max}$	MEM 2 BCA	MEM 4 SESA$_{max}$	MEM 4 BCA	MEM 8 SESA$_{max}$	MEM 8 BCA	MEM 16 SESA$_{max}$	MEM 16 BCA	MEM 32 SESA$_{max}$	MEM 32 BCA	MEM 64 SESA$_{max}$	MEM 64 BCA
0	5.7	9.0	5.7	9.0	6.2	9.0	6.2	9.0	6.3	9.0	6.3	9.0	6.3	8.9	6.4	8.9	6.3	8.9	6.2	9.0	6.3	8.9	5.4	8.6
AVI 0.062	5.6	9.0	6.2	9.0	6.2	9.0	6.3	9.0	6.3	9.0	6.3	8.9	6.4	8.9	6.3	8.8	6.4	8.9	5.8	8.6	5.7	8.7	5.2	7.9
AVI 0.125	5.8	9.0	6.2	9.0	6.2	9.0	6.2	9.0	6.3	8.9	6.4	8.9	6.4	8.9	6.3	8.9	6.4	8.9	5.8	8.6	5.5	7.9	5.0	7.6
AVI 0.25	5.9	9.0	6.2	9.0	6.3	9.0	6.3	9.0	6.3	8.9	6.4	8.8	6.3	8.8	6.2	8.4	6.0	7.9	5.8	8.4	5.1	8.8	5.2	7.8
AVI 0.5	5.4	9.0	6.2	9.0	6.3	9.0	6.3	8.9	6.4	8.8	6.4	8.7	6.3	8.5	6.2	8.3	5.8	7.8	5.8	8.8	5.1	8.8	5.1	8.0
AVI 1	5.9	9.0	6.2	9.0	6.3	9.0	6.4	8.9	6.4	8.7	6.3	8.5	6.2	8.5	6.0	8.3	5.6	7.8	5.2	8.1	4.8	8.8	4.7	6.9
AVI 2	6.1	9.0	6.3	8.9	6.4	8.8	6.4	8.8	6.2	8.2	5.4	7.5	ND		ND		ND		ND		ND		ND	
AVI 4	6.3	8.9	6.4	8.9	6.4	8.8	6.4	8.6	5.8	7.4	5.4	7.4	ND		ND		ND		ND		ND		ND	

KPC-2 ΔOmpK35/36 — Viable count (log_{10} CFU/mL)

AVI	0	MEM 0.062	MEM 0.125	MEM 0.25	MEM 0.5	MEM 1	MEM 2	MEM 4	MEM 8	MEM 16	MEM 32	MEM 64
0	9	9	9	9	8	8	8	8	8	8	7	1
AVI 0.062	8	7	8	8	7	8	7	7	7	1	1	1
AVI 0.125	9	7	7	8	7	7	8	7	6	1	1	1
AVI 0.25	8	7	7	7	6	6	7	1	1	1	1	1
AVI 0.5	9	7	7	7	7	1	1	1	1	1	1	1
AVI 1	7	7	6	6	1	1	1	1	1	1	1	1
AVI 2	9	7	7	1	1	1	ND	ND	ND	ND	ND	ND
AVI 4	7	6	7	1	1	1	ND	ND	ND	ND	ND	ND

Abbreviations: AVI, avibactam; MEM, meropenem; ND, not determined.

(C)

OXA-48 — **Time-lapse microscopy**

	0		MEM 0.016		MEM 0.031		MEM 0.062		MEM 0.125		MEM 0.25		MEM 0.5		MEM 1		MEM 2		MEM 4		MEM 8	
	$SESA_{max}$	BCA	$SESA_{max}$	BCA	$SESA_{max}$	BCA	$SESA_{max}$	BCA	$SESA_{max}$	BCA	$SESA_{max}$	BCA	$SESA_{max}$	BCA	$SESA_{max}$	BCA	$SESA_{max}$	BCA	$SESA_{max}$	BCA	$SESA_{max}$	BCA
0	6.2	8.9	6.3	8.9	6.3	8.9	6.3	9.0	6.0	7.6	6.0	7.7	5.7	7.1	5.2	6.2	4.7	5.6	4.5	5.5	5.0	5.3
AVI 0.062	6.1	9.0	6.3	8.9	6.3	8.9	6.1	7.9	5.9	7.7	6.0	7.4	5.5	7.0	5.3	6.9	4.9	6.5	4.7	5.4	4.5	5.6
AVI 0.125	6.1	9.0	6.3	8.9	6.3	8.9	6.0	7.9	5.9	7.7	6.0	7.4	5.9	7.2	5.2	6.7	5.1	6.6	4.6	6.0	4.7	6.0
AVI 0.25	6.0	9.0	6.3	8.9	6.3	8.8	6.0	7.7	6.1	7.5	5.9	7.4	5.7	7.2	5.3	6.6	5.2	6.6	5.1	7.0	4.5	6.3
AVI 0.5	6.2	8.9	6.3	8.9	6.2	8.3	6.1	7.9	5.9	7.7	6.0	7.3	5.9	7.1	5.3	6.9	5.0	5.9	4.8	7.2	4.3	5.9
AVI 1	6.3	8.8	6.3	8.8	6.3	8.3	5.9	7.9	6.1	7.8	5.9	7.2	5.5	7.0	5.4	6.4	5.1	7.0	4.4	5.7	4.7	5.8
AVI 2	6.1	9.0	6.4	8.9	5.6	8.6	5.3	8.5	6.3	8.9	ND		ND		ND		ND		ND		ND	
AVI 4	6.2	9.0	6.3	8.9	5.7	8.5	5.3	8.4	5.3	8.1	ND		ND		ND		ND		ND		ND	

OXA-48 — **Viable count** (log_{10} CFU/mL)

	0	MEM 0.016	MEM 0.031	MEM 0.062	MEM 0.125	MEM 0.25	MEM 0.5	MEM 1	MEM 2	MEM 4	MEM 8
0	8	7	7	7	1	1	1	1	1	1	1
AVI 0.062	8	7	7	1	1	1	1	1	1	1	1
AVI 0.125	9	8	7	1	1	1	1	1	1	1	1
AVI 0.25	9	8	7	1	1	1	1	1	1	1	1
AVI 0.5	8	7	1	1	1	1	1	1	1	1	1
AVI 1	8	7	1	1	1	1	1	1	1	1	1
AVI 2	8	6	1	1	7	ND	ND	ND	ND	ND	ND
AVI 4	7	7	1	1	1	ND	ND	ND	ND	ND	ND

OXA-48 ΔOmpK35/36 — **Time-lapse microscopy**

	0		MEM 0.031		MEM 0.062		MEM 0.125		MEM 0.25		MEM 0.5		MEM 1		MEM 2		MEM 4		MEM 8		MEM 16		MEM 32	
	$SESA_{max}$	BCA	$SESA_{max}$	BCA	$SESA_{max}$	BCA	$SESA_{max}$	BCA	$SESA_{max}$	BCA	$SESA_{max}$	BCA	$SESA_{max}$	BCA	$SESA_{max}$	BCA	$SESA_{max}$	BCA	$SESA_{max}$	BCA	$SESA_{max}$	BCA	$SESA_{max}$	BCA
0	5.6	9.0	5.8	9.0	5.8	9.0	6.0	9.0	5.7	9.0	6.1	9.0	6.2	9.0	6.3	8.9	6.4	8.9	5.3	6.6	4.9	6.0	4.9	6.4
AVI 0.062	5.8	9.0	6.0	9.0	5.8	9.0	6.0	9.0	5.9	9.0	6.2	9.0	6.3	8.9	6.3	8.9	6.3	8.8	5.5	6.9	5.2	7.0	4.6	6.5
AVI 0.125	6.0	9.0	6.0	9.0	5.9	9.0	6.0	9.0	6.2	9.0	6.3	8.9	6.3	8.9	6.4	8.8	6.3	8.9	5.3	6.6	4.6	6.5	5.1	7.0
AVI 0.25	5.9	9.0	6.0	9.0	6.1	9.0	6.1	9.0	6.2	9.0	6.3	8.9	6.4	8.9	6.4	8.8	6.0	7.2	5.4	7.2	5.2	6.7	5.2	6.7
AVI 0.5	5.9	9.0	6.2	9.0	6.2	9.0	6.3	9.0	6.2	8.9	6.3	8.9	6.4	8.8	6.1	7.9	5.6	7.1	5.5	6.8	5.0	6.9	4.8	5.8
AVI 1	5.6	9.0	6.3	8.9	6.2	9.0	6.2	8.9	6.3	8.9	6.4	8.8	6.3	8.5	6.2	7.6	5.3	6.3	5.1	6.8	4.9	6.6	4.3	5.9
AVI 2	6.2	9.0	6.3	9.0	6.3	8.9	6.3	8.9	6.4	8.8	5.9	8.6	5.4	8.8	ND		ND		ND		ND		ND	
AVI 4	6.3	9.0	6.3	8.9	6.3	8.9	6.4	8.9	6.2	8.7	5.7	8.8	5.2	8.8	ND		ND		ND		ND		ND	

OXA-48 ΔOmpK35/36 — **Viable count** (log_{10} CFU/mL)

	0	MEM 0.031	MEM 0.062	MEM 0.125	MEM 0.25	MEM 0.5	MEM 1	MEM 2	MEM 4	MEM 8	MEM 16	MEM 32
0	9	8	8	7	8	8	8	8	7	1	1	1
AVI 0.062	7	8	7	7	7	7	7	7	7	1	1	1
AVI 0.125	7	8	7	8	7	7	7	7	7	1	1	1
AVI 0.25	7	8	8	7	7	7	7	7	1	1	1	1
AVI 0.5	8	7	8	8	7	7	5	1	1	1	1	1
AVI 1	8	8	8	7	7	7	1	1	1	1	1	1
AVI 2	8	7	7	7	6	1	1	ND	ND	ND	ND	ND
AVI 4	7	6	6	6	1	1	1	ND	ND	ND	ND	ND

Abbreviations: AVI, avibactam; MEM, meropenem; ND, not determined.

		Ceftazidime			Meropenem		
			$SESA_{max}$	BCA		$SESA_{max}$	BCA
**Parental strain**	**Time-lapse microscopy**	0	5.7	9.0	0	5.8	9.0
		CAZ 0.062	6.3	9.0	MEM 0.0078	6.0	9.0
		CAZ 0.125	6.3	9.0	MEM 0.016	6.4	8.9
		CAZ 0.25	6.2	8.2	MEM 0.031	6.3	8.5
		CAZ 0.5	6.2	8.2	MEM 0.062	6.3	8.3
		CAZ 1	6.2	8.1	MEM 0.125	6.2	8.3
		CAZ 2	6.2	8.0	MEM 0.25	5.9	8.2
		CAZ 4	6.0	7.4	MEM 0.5	5.2	8.1
	**Viable count** (log_{10} CFU/mL)	0	9		0	9	
		CAZ 0.062	8		MEM 0.0078	8	
		CAZ 0.125	8		MEM 0.016	7	
		CAZ 0.25	1		MEM 0.031	1	
		CAZ 0.5	1		MEM 0.062	1	
		CAZ 1	1		MEM 0.125	1	
		CAZ 2	1		MEM 0.25	1	
		CAZ 4	1		MEM 0.5	1	

		Ceftazidime			Meropenem		
			$SESA_{max}$	BCA		$SESA_{max}$	BCA
**ΔOmpK35/36 strain**	**Time-lapse microscopy**	0	5.8	9.0	0	5.6	9.0
		CAZ 0.062	5.8	9.0	MEM 0.0078	5.8	9.0
		CAZ 0.125	6.3	9.0	MEM 0.016	6.2	9.0
		CAZ 0.25	6.0	8.0	MEM 0.031	6.4	8.9
		CAZ 0.5	6.0	7.4	MEM 0.062	6.4	8.6
		CAZ 1	5.9	7.0	MEM 0.125	6.2	8.3
		CAZ 2	5.4	7.0	MEM 0.25	6.2	7.8
		CAZ 4	5.3	6.8	MEM 0.5	5.7	7.4
	**Viable count** (log_{10} CFU/mL)	0	9		0	8	
		CAZ 0.062	9		MEM 0.0078	8	
		CAZ 0.125	8		MEM 0.016	7	
		CAZ 0.25	1		MEM 0.031	7	
		CAZ 0.5	1		MEM 0.062	4	
		CAZ 1	1		MEM 0.125	1	
		CAZ 2	1		MEM 0.25	1	
		CAZ 4	1		MEM 0.5	1	

Abbreviations: CAZ, ceftazidime; MEM, meropenem.

Figure S2. Automated time-lapse microscopy readouts and viable counts (log_{10} CFU/mL) from screening with ceftazidime and meropenem (mg/L) against the *K. pneumoniae* ATCC 35657 parental and ΔOmpK35/36 strains. The 24-hour BCA and $SESA_{max}$ values are presented, and values above the predefined cut-offs (BCA > 8.0, $SESA_{max}$ > 5.8) are marked in grey. For viable counts, no visible growth was recorded as 1 log_{10} CFU/mL. Bacterial densities > 6 log_{10} CFU/mL are marked in grey.

(A)

															Avibactam fixed at 0.125 mg/L										Avibactam fixed at 0.5 mg/L									
			0		CAZ 0.125		CAZ 0.25		CAZ 0.5		CAZ 1		CAZ 4		CAZ 0.125		CAZ 0.25		CAZ 0.5		CAZ 1		CAZ 4		CAZ 0.125		CAZ 0.25		CAZ 0.5		CAZ 1		CAZ 4	
			$SESA_{max}$	BCA	$SESA_{max}$	BCA	$SESA_{max}$	BCA	$SESA_{max}$	BCA	$SESA_{max}$	BCA	$SESA_{max}$	BCA	$SESA_{max}$	BCA	$SESA_{max}$	BCA	$SESA_{max}$	BCA	$SESA_{max}$	BCA	$SESA_{max}$	BCA	$SESA_{max}$	BCA	$SESA_{max}$	BCA	$SESA_{max}$	BCA	$SESA_{max}$	BCA	$SESA_{max}$	BCA
**CTX-M-15**	**Time-lapse microscopy**	0	5.7	9.0	5.9	9.0	5.7	9.0	5.8	9.0	5.9	9.0	5.9	9.0	6.3	8.9	6.3	8.8	6.3	8.2	6.3	8.1	6.2	8.0	6.3	8.8	6.2	8.3	6.2	8.2	6.3	8.3	6.2	8.0
		COL 0.062	5.7	9.0	6.0	9.0	6.1	9.0	5.9	9.0	6.0	9.0	5.9	9.0	6.2	8.9	6.3	8.8	6.2	8.2	6.2	8.1	6.2	8.1	6.3	8.9	6.3	8.4	6.2	8.2	6.0	8.1	6.1	8.3
		COL 0.125	6.0	9.0	6.1	9.0	6.0	9.0	6.1	9.0	6.0	9.0	6.1	9.0	6.2	8.9	6.3	8.8	6.2	8.1	5.7	7.7	5.5	7.7	6.3	8.9	6.2	8.3	6.0	8.2	5.7	7.8	5.8	8.2
		COL 0.25	4.7	8.9	4.6	8.8	4.6	8.8	4.8	8.8	4.7	8.8	6.3	9.0	4.6	7.4	4.9	6.6	4.6	6.5	4.5	6.5	4.7	6.9	6.3	8.8	5.2	7.1	5.5	7.9	4.8	8.5	5.1	8.5
		COL 0.5	4.4	8.9	4.8	8.9	4.4	8.8	4.4	8.8	4.6	8.8	6.3	9.0	4.8	8.1	4.8	7.0	4.7	6.9	4.4	6.7	4.5	7.1	5.0	7.1	4.6	7.7	4.5	7.9	4.5	7.9	4.9	8.5
		COL 1	4.6	7.0	4.7	6.8	4.6	8.8	4.6	8.7	4.8	7.8	4.8	6.8	4.8	8.3	4.6	7.5	4.6	7.1	4.5	6.8	4.7	6.5	4.9	7.0	4.7	6.8	4.5	8.3	5.0	7.4	4.8	6.9
	**Viable count** (log_{10} CFU/mL)	0	8		7		8		8		8		7		8		6		1		1		1		3		1		1		1		1	
		COL 0.062	8		8		8		8		8		7		7		3		1		1		1		7		1		1		1		1	
		COL 0.125	8		8		8		8		8		7		8		7		1		1		1		6		1		1		1		1	
		COL 0.25	1		1		1		1		1		7		1		1		1		1		1		7		1		1		1		1	
		COL 0.5	1		1		1		1		1		7		1		1		1		1		1		1		1		1		1		1	
		COL 1	1		1		1		1		1				1		1		1		1		1		1		1		1		1		1	

															Avibactam fixed at 0.125 mg/L										Avibactam fixed at 0.5 mg/L									
			0		CAZ 0.5		CAZ 1		CAZ 2		CAZ 4		CAZ 16		CAZ 0.5		CAZ 1		CAZ 2		CAZ 4		CAZ 16		CAZ 0.5		CAZ 1		CAZ 2		CAZ 4		CAZ 16	
			$SESA_{max}$	BCA	$SESA_{max}$	BCA	$SESA_{max}$	BCA	$SESA_{max}$	BCA	$SESA_{max}$	BCA	$SESA_{max}$	BCA	$SESA_{max}$	BCA	$SESA_{max}$	BCA	$SESA_{max}$	BCA	$SESA_{max}$	BCA	$SESA_{max}$	BCA	$SESA_{max}$	BCA	$SESA_{max}$	BCA	$SESA_{max}$	BCA	$SESA_{max}$	BCA	$SESA_{max}$	BCA
**CTX-M-15 ΔOmpK35/36**	**Time-lapse microscopy**	0	5.3	9.0	5.4	9.0	5.6	9.0	5.6	9.0	6.0	9.0	6.2	9.0	5.9	9.0	5.9	9.0	6.1	9.0	6.2	8.9	6.3	8.9	6.3	8.7	6.0	8.1	5.8	7.8	6.3	8.9	5.3	7.4
		COL 0.062	5.5	9.0	5.5	9.0	5.9	9.0	5.7	9.0	6.0	9.0	6.2	9.0	5.8	9.0	6.0	9.0	6.2	9.0	6.2	8.9	6.3	8.7	6.3	8.8	6.1	8.1	5.9	8.0	5.9	8.3	5.5	7.2
		COL 0.125	5.6	9.0	6.0	9.0	5.9	9.0	6.1	9.0	6.1	9.0	6.3	9.0	6.1	9.0	6.2	9.0	6.2	8.9	6.2	8.9	4.9	8.8	6.3	8.8	6.0	8.5	5.5	7.9	5.5	8.6	5.0	7.8
		COL 0.25	4.7	8.9	6.2	9.0	4.6	8.8	4.5	8.8	4.8	8.7	4.9	8.6	6.3	9.0	4.6	7.6	6.3	8.9	4.9	7.4	4.7	8.8	4.9	7.7	5.1	7.6	4.9	7.4	4.7	8.5	4.6	8.8
		COL 0.5	5.8	9.0	4.6	8.8	4.8	8.8	4.6	8.8	4.9	8.7	4.7	8.6	5.0	8.1	5.0	7.2	4.7	6.9	4.9	6.8	4.8	7.1	5.0	7.1	4.9	7.3	4.7	7.8	4.5	8.7	4.4	8.8
		COL 1	4.5	8.9	4.7	8.9	4.6	8.8	4.7	8.8	4.7	8.8	4.7	8.4	5.1	7.8	5.0	7.4	4.9	7.7	5.0	7.6	4.4	8.8	4.9	7.2	4.8	8.6	4.6	7.4	4.9	7.7	4.7	8.2
	**Viable count** (log_{10} CFU/mL)	0	9		9		9		8		8		7		8		8		8		7		7		6		5		1		1		1	
		COL 0.062	9		9		9		8		8		7		8		7		8		7		6		7		5		1		1		1	
		COL 0.125	9		8		8		8		8		8		8		8		7		7		1		6		6		1		1		1	
		COL 0.25	1		8		1		1		1		1		8		1		7		1		1		1		1		1		1		1	
		COL 0.5	7		1		1		1		1		1		1		1		1		1		1		1		1		1		1		1	
		COL 1	1		1		1		1		1		1		1		1		1		1		1		1		1		1		1		1	

Abbreviations: AVI, avibactam; CAZ, ceftazidime; COL, colistin.

Figure S3. Automated time-lapse microscopy readouts and viable counts (log_{10} CFU/mL) from screening of antibiotic combination effects against *K. pneumoniae* ATCC 35657 constructed strains, **(A)** CTX-M-15, and CTX-M-15 ΔOmpK35/36 exposed to COL (mg/L) in combination with CAZ and CAZ-AVI (mg/L), **(B)** KPC-2, or and KPC-2 ΔOmpK35/36 exposed to COL (mg/L) in combination with MEM and MEM-AVI (mg/L), **(C)** OXA-48 and OXA-48 ΔOmpK35/36 exposed to COL (mg/L) in combination with MEM and MEM-AVI (mg/L). The 24-hour BCA and $SESA_{max}$ values are presented, and values above the predefined cut-offs (BCA > 8.0, $SESA_{max}$ > 5.8) are marked in grey. For viable counts, no visible growth is marked as 1 log_{10} CFU/mL, and bacterial density > 6 log_{10} CFU/mL is marked in grey.

(B)

Strain	Method	COL	0		MEM 0.0078		MEM 0.016		MEM 0.031		MEM 0.062		MEM 0.25		Avibactam fixed at 0.125 mg/L: MEM 0.0078		MEM 0.016		MEM 0.031		MEM 0.062		MEM 0.25		Avibactam fixed at 0.5 mg/L: MEM 0.0078		MEM 0.016		MEM 0.031		MEM 0.062		MEM 0.25	
			$SESA_{max}$	BCA	$SESA_{max}$	BCA	$SESA_{max}$	BCA	$SESA_{max}$	BCA	$SESA_{max}$	BCA	$SESA_{max}$	BCA	$SESA_{max}$	BCA	$SESA_{max}$	BCA	$SESA_{max}$	BCA	$SESA_{max}$	BCA	$SESA_{max}$	BCA	$SESA_{max}$	BCA	$SESA_{max}$	BCA	$SESA_{max}$	BCA	$SESA_{max}$	BCA	$SESA_{max}$	BCA
KPC-2	Time-lapse microscopy	0	6.1	9.0	6.2	9.0	6.2	9.0	6.3	8.9	6.3	8.9	6.3	9.0	6.3	8.9	6.4	8.9	6.3	8.9	6.3	8.3	6.1	8.1	6.3	8.9	6.4	8.9	6.3	8.6	6.2	8.3	5.9	7.8
		COL 0.062	5.8	9.0	5.9	9.0	6.2	9.0	6.2	9.0	6.3	8.9	6.2	8.9	6.3	8.9	6.3	8.9	6.3	8.9	6.0	8.4	5.9	8.0	6.3	8.9	6.4	8.9	5.3	7.6	6.1	8.3	5.6	7.5
		COL 0.125	5.9	9.0	6.1	9.0	6.2	9.0	6.3	9.0	6.3	9.0	6.3	9.0	6.3	8.9	6.3	8.9	5.7	8.3	5.6	7.6	5.3	7.5	6.3	8.9	6.3	8.8	4.9	8.4	5.5	8.7	5.3	7.5
		COL 0.25	6.3	9.0	6.2	9.0	6.3	9.0	6.3	9.0	6.3	9.0	5.2	7.1	6.3	9.0	6.3	8.9	5.3	8.7	4.6	8.0	4.9	7.5	6.3	8.9	5.2	8.2	5.3	8.6	4.7	8.8	5.2	8.8
		COL 0.5	5.3	6.8	4.9	6.3	6.3	9.0	5.0	6.8	5.3	7.1	5.4	7.3	4.7	7.2	4.7	8.5	4.5	8.2	4.5	7.2	4.5	7.6	4.6	7.2	6.3	8.9	5.3	8.7	5.0	8.8	5.9	8.8
		COL 1	5.3	6.9	4.8	6.2	4.7	6.5	4.8	6.6	5.2	7.0	5.2	6.7	5.3	6.9	4.7	6.6	4.7	8.2	4.7	7.1	4.4	6.8	4.5	7.0	4.3	7.8	4.7	8.7	4.4	8.8	3.8	8.8
	Viable count (log_{10} CFU/mL)	0	8		8		8		8		8		8		8		8		8		1		1		8		7		6		1		1	
		COL 0.062	9		8		9		9		8		8		9		8		7		4		1		7		7		1		2		1	
		COL 0.125	8		8		9		8		8		8		8		7		3		1		1		8		7		1		1		1	
		COL 0.25	8		8		8		8		8		1		8		7		1		1		1		7		1		1		1		1	
		COL 0.5	1		6		7		1		1		1		1		1		1		1		1		1		7		1		1		1	
		COL 1	1		1		1		1		1		1		1		1		1		1		1		1		1		1		1		1	

Strain	Method	COL	0		MEM 0.25		MEM 1		MEM 2		MEM 4		MEM 16		Avibactam fixed at 0.125 mg/L: MEM 0.25		MEM 1		MEM 2		MEM 4		MEM 16		Avibactam fixed at 0.5 mg/L: MEM 0.25		MEM 1		MEM 2		MEM 4		MEM 16	
			$SESA_{max}$	BCA	$SESA_{max}$	BCA	$SESA_{max}$	BCA	$SESA_{max}$	BCA	$SESA_{max}$	BCA	$SESA_{max}$	BCA	$SESA_{max}$	BCA	$SESA_{max}$	BCA	$SESA_{max}$	BCA	$SESA_{max}$	BCA	$SESA_{max}$	BCA	$SESA_{max}$	BCA	$SESA_{max}$	BCA	$SESA_{max}$	BCA	$SESA_{max}$	BCA	$SESA_{max}$	BCA
KPC-2 ΔOmpK35/36	Time-lapse microscopy	0	5.8	9.0	5.7	9.0	6.3	9.0	6.3	9.0	6.3	9.0	6.3	9.0	6.3	8.9	6.4	8.8	6.4	8.8	6.3	8.9	5.4	8.1	6.4	8.9	6.3	8.6	6.1	8.3	5.8	7.7	5.1	6.8
		COL 0.062	5.9	9.0	5.9	9.0	6.3	9.0	6.3	8.9	6.3	8.9	6.3	9.0	6.3	8.9	6.4	8.8	6.3	8.9	5.8	8.5	5.2	8.6	6.3	8.9	6.1	8.6	6.0	8.6	5.8	8.3	5.1	8.1
		COL 0.125	6.0	9.0	5.9	9.0	6.3	9.0	6.3	9.0	6.3	9.0	6.3	8.9	6.3	8.9	6.3	8.8	5.7	8.7	5.4	8.5	4.8	8.8	6.4	8.9	5.9	8.6	5.7	8.7	5.5	8.0	5.3	7.7
		COL 0.25	6.3	9.0	6.3	9.0	6.3	8.9	5.1	6.8	4.7	6.5	5.2	6.9	6.3	8.9	4.5	8.5	4.9	8.6	4.8	8.5	4.9	8.4	5.1	8.8	4.7	8.8	5.0	8.8	5.4	8.6	5.0	8.4
		COL 0.5	6.3	8.9	4.2	6.7	5.0	6.7	4.8	6.5	4.8	6.4	5.0	7.2	4.8	7.2	4.6	8.7	4.5	8.5	4.7	8.2	4.4	7.1	4.8	8.7	5.2	8.7	5.0	8.8	5.0	8.6	5.0	6.9
		COL 1	4.4	7.2	5.2	7.4	5.2	7.2	4.7	6.8	4.9	6.4	5.0	6.8	4.9	6.8	4.8	7.6	4.8	8.1	5.3	8.2	5.0	8.2	4.4	8.8	5.1	8.8	4.6	8.8	4.8	8.2	4.6	8.0
	Viable count (log_{10} CFU/mL)	0	8		8		8		8		9		8		8		7		7		6		1		7		2		1		1		1	
		COL 0.062	9		8		7		8		8		8		9		7		7		1		1		7		1		1		1		1	
		COL 0.125	8		8		8		7		7		7		9		7		1		2		1		6		1		1		1		1	
		COL 0.25	8		7		8		1		1		1		8		1		1		1		1		1		1		1		1		1	
		COL 0.5	7		1		1		1		1		1		1		1		1		1		1		1		1		1		1		1	
		COL 1	1		1		1		1		1		1		1		1		1		1		1		1		1		1		1		1	

Abbreviations: AVI, avibactam; CAZ, ceftazidime; COL, colistin.

(C)

															Avibactam fixed at 0.125 mg/L										Avibactam fixed at 0.5 mg/L									
			0		MEM 0.0078		MEM 0.016		MEM 0.031		MEM 0.062		MEM 0.125		MEM 0.0078		MEM 0.016		MEM 0.031		MEM 0.062		MEM 0.125		MEM 0.0078		MEM 0.016		MEM 0.031		MEM 0.062		MEM 0.125	
			$SESA_{max}$	BCA	$SESA_{max}$	BCA	$SESA_{max}$	BCA	$SESA_{max}$	BCA	$SESA_{max}$	BCA	$SESA_{max}$	BCA	$SESA_{max}$	BCA	$SESA_{max}$	BCA	$SESA_{max}$	BCA	$SESA_{max}$	BCA	$SESA_{max}$	BCA	$SESA_{max}$	BCA	$SESA_{max}$	BCA	$SESA_{max}$	BCA	$SESA_{max}$	BCA	$SESA_{max}$	BCA
**OXA-48**	**Time-lapse microscopy**	0	6.0	9.0	5.9	9.0	6.3	8.9	6.3	8.9	6.3	9.0	5.7	7.6	6.2	9.0	6.4	8.9	6.3	8.8	6.2	8.0	6.1	7.8	6.3	8.9	6.4	8.9	6.3	8.4	5.9	8.0	5.5	8.3
		COL 0.062	6.0	9.0	6.0	9.0	6.2	8.9	6.3	8.9	6.2	9.0	5.8	7.4	6.2	9.0	6.4	8.9	6.3	8.3	6.0	7.8	5.8	7.6	6.3	8.9	6.3	8.9	6.1	8.5	5.7	8.1	5.6	7.7
		COL 0.125	6.1	9.0	6.1	9.0	6.3	8.9	6.2	8.9	5.5	7.3	5.2	7.0	6.3	9.0	6.3	9.0	4.7	7.0	4.7	6.6	5.1	6.8	6.3	8.9	5.6	8.7	5.5	8.7	5.7	8.8	5.2	7.5
		COL 0.25	6.3	9.0	6.3	9.0	6.3	9.0	4.7	6.3	4.7	6.2	5.0	6.2	4.8	8.7	4.7	8.0	4.5	8.0	4.7	6.8	4.7	6.6	5.2	8.6	5.1	8.8	4.4	8.8	5.0	8.8	5.0	8.8
		COL 0.5	4.8	7.1	5.0	6.3	4.5	6.8	4.7	6.7	4.6	6.6	5.0	6.8	4.8	8.6	4.6	7.0	4.9	7.3	4.8	7.1	4.8	7.1	4.6	8.6	4.6	8.7	4.8	8.7	4.4	8.8	4.9	8.8
		COL 1	4.6	6.9	4.8	6.5	5.0	7.0	4.6	6.7	4.9	6.6	5.0	6.8	4.9	8.7	4.5	8.6	5.2	8.7	4.8	8.6	5.4	8.6	4.8	8.5	4.6	8.2	4.3	8.8	4.3	8.8	4.6	8.9
	**Viable count** (log_{10} CFU/mL)	0	8		8		8		8		8		1		7		7		7		2		1		8		7		1		1		1	
		COL 0.062	9		8		8		8		7		1		8		7		1		1		1		7		7		1		1		1	
		COL 0.125	9		8		8		8		1		1		8		7		1		1		1		7		1		1		1		1	
		COL 0.25	9		8		8		1		1		1		1		2		1		1		1		1		1		1		1		1	
		COL 0.5	1		1		1		1		1		1		1		1		1		1		1		1		1		1		1		1	
		COL 1	1		1		1		1		1		1		1		1		1		1		1		1		1		1		1		1	

			0		MEM 0.125		MEM 0.5		MEM 1		MEM 2		MEM 4		MEM 0.125		MEM 0.5		MEM 1		MEM 2		MEM 4		MEM 0.125		MEM 0.5		MEM 1		MEM 2		MEM 4	
			$SESA_{max}$	BCA	$SESA_{max}$	BCA	$SESA_{max}$	BCA	$SESA_{max}$	BCA	$SESA_{max}$	BCA	$SESA_{max}$	BCA	$SESA_{max}$	BCA	$SESA_{max}$	BCA	$SESA_{max}$	BCA	$SESA_{max}$	BCA	$SESA_{max}$	BCA	$SESA_{max}$	BCA	$SESA_{max}$	BCA	$SESA_{max}$	BCA	$SESA_{max}$	BCA	$SESA_{max}$	BCA
**OXA-48 ΔOmpK35/36**	**Time-lapse microscopy**	0	5.8	9.0	5.9	9.0	5.9	9.0	6.1	9.0	6.3	9.0	6.3	8.9	5.9	9.0	6.0	9.0	6.3	8.9	6.4	8.9	5.3	6.4	6.0	9.0	6.4	8.9	6.4	8.8	5.5	7.4	4.8	6.0
		COL 0.062	6.0	9.0	5.9	9.0	6.0	9.0	6.1	9.0	6.3	9.0	6.3	8.9	5.7	9.0	6.1	9.0	6.3	8.9	6.3	8.9	5.2	7.0	6.0	9.0	6.4	8.9	6.4	8.8	5.6	7.5	5.3	6.9
		COL 0.125	6.1	9.0	6.1	9.0	6.1	9.0	6.1	9.0	6.2	9.0	4.8	8.8	6.0	9.0	6.2	9.0	6.3	8.9	6.3	8.9	4.9	6.8	6.2	9.0	6.3	8.9	5.3	7.1	5.2	7.1	5.3	7.8
		COL 0.25	5.0	8.9	4.8	8.9	4.9	8.8	6.3	9.0	6.3	9.0	4.5	8.8	6.2	9.0	6.3	8.9	5.1	7.5	5.1	7.2	5.0	7.0	4.6	6.5	4.9	7.2	5.0	7.3	4.8	8.0	5.0	8.7
		COL 0.5	5.0	8.9	4.9	8.8	5.1	8.9	5.1	8.8	4.6	8.6	4.9	7.9	4.9	8.3	4.7	8.2	5.1	7.8	4.6	7.0	5.0	7.4	4.8	6.7	4.9	7.0	4.7	6.8	4.7	7.1	4.8	8.6
		COL 1	4.9	8.9	5.0	8.8	5.1	7.8	5.0	8.0	5.0	8.9	4.9	8.8	6.3	8.8	4.4	7.8	4.9	7.8	4.6	7.1	5.1	7.3	5.1	6.9	5.1	7.3	4.9	8.0	5.0	6.8	5.1	6.7
	**Viable count** (log_{10} CFU/mL)	0	8		8		7		7		7		7		9		9		8		7		1		8		7		7		1		1	
		COL 0.062	8		8		8		7		7		7		8		8		7		7		1		8		8		7		1		1	
		COL 0.125	8		8		8		7		7		1		8		8		7		7		1		7		8		1		1		1	
		COL 0.25	1		1		1		7		7		1		8		8		1		1		1		1		1		1		1		1	
		COL 0.5	1		3		1		1		6		1		4		1		1		1		1		1		1		1		1		1	
		COL 1	1		1		1		1		1		1		8		1		1		1		1		1		1		1		1		1	

Abbreviations: AVI, avibactam; CAZ, ceftazidime; COL, colistin.

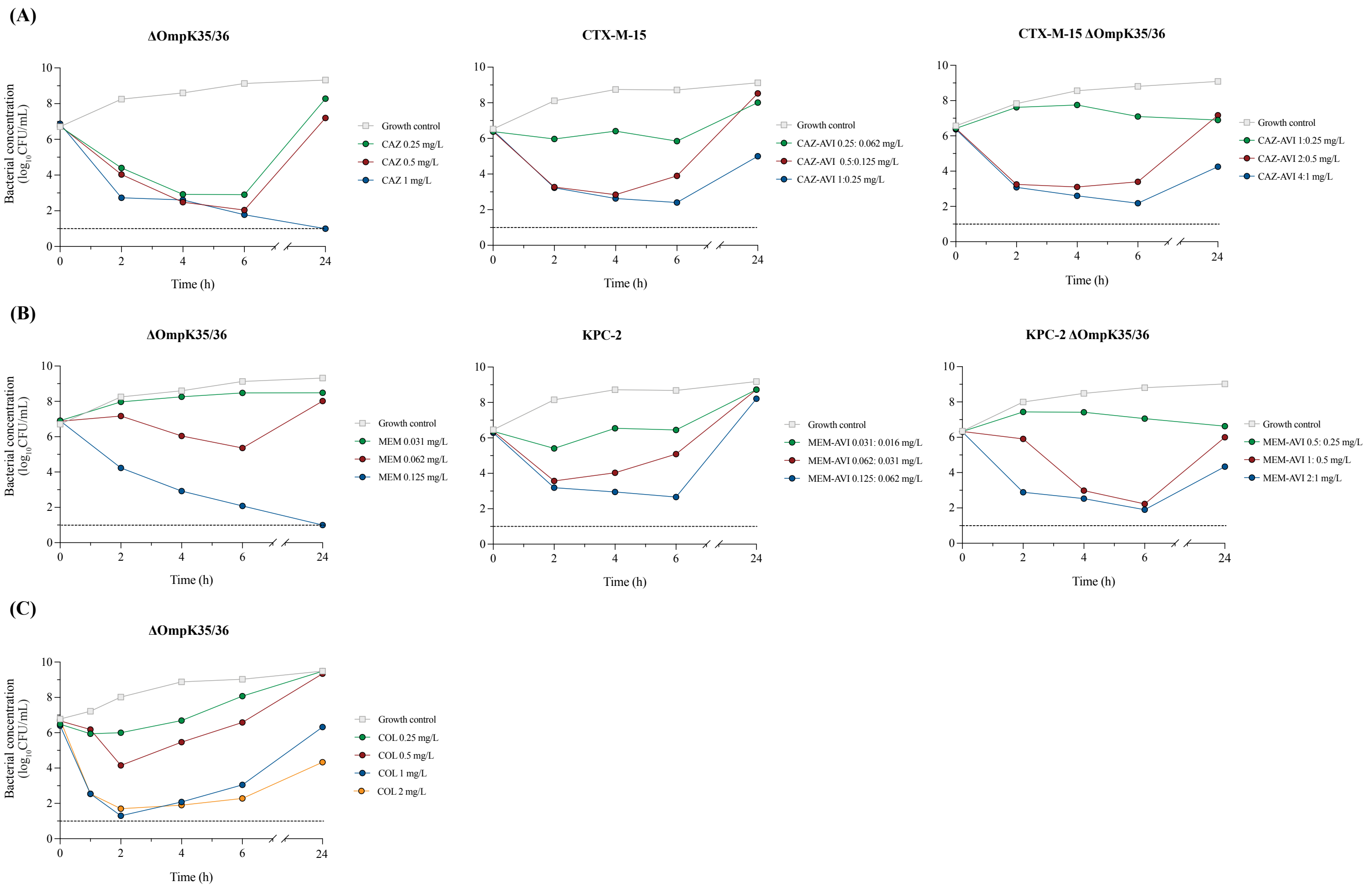

Abbreviations: CAZ, ceftazidime; CAZ-AVI, ceftazidime-avibactam; COL, colistin; MEM, meropenem; MEM-AVI, meropenem-avibactam.

Figure S4. Time-kill experiments with *K. pneumoniae* ATCC 35657 constructs with **(A)** ceftazidime or ceftazidime-avibactam at 0.5x, 1x, and 2x MIC or MIC_{ratio}, respectively, **(B)** meropenem or meropenem-avibactam at 0.5x, 1x, and 2x MIC or MIC_{ratio}, respectively **(C)** colistin at 1x, 2x, 4x, and 8x MIC. LOD (1 log_{10} CFU/mL) is marked with a dotted line.
